# *iNOS* Mediates High-Fat Diet-Associated Aggravation of Complete Freund’s Adjuvant-Induced Inflammatory Pain

**DOI:** 10.3390/ijms26115422

**Published:** 2025-06-05

**Authors:** Elmo Wing-Yiu Lee, Lin Wang, Jessica Ai-Jia Liu, Chi-Wai Cheung

**Affiliations:** 1 Department of Anaesthesiology, School of Clinical Medicine, LKS Faculty of Medicine, The University of Hong Kong, Hong Kong SAR, China; u3569846@connect.hku.hk (E.W.-Y.L.); rachelew@connect.hku.hk (L.W.); 2 Department of Neuroscience, City University of Hong Kong, Hong Kong SAR, China; 3 Hong Kong Sanatorium & Hospital, Hong Kong SAR, China

**Keywords:** inflammatory pain, short-term high-fat diet, obesity, *iNOS*

## Abstract

Chronic inflammatory pain (IP) remains a therapeutic challenge under the worldwide prevalence of the high-fat dietary lifestyle. This study aimed at identifying mediators of the IP augmented by short-term high-fat diet (HFD). IP was induced on C57BL/6J mice by unilateral, intra-plantar, injection of Complete Freund’s Adjuvant (CFA). Von Frey test for mechanical hyperalgesia and Hargreaves’ test for thermal hyperalgesia were performed at pre-injection baseline and post-injection 6th h. and days 1/3/5/7/10/14. Ad libitum HFD feeding started 2 weeks pre-injection in assigned groups. Body weight and random blood glucose levels were measured. RT-qPCR and ELISA helped quantify expression levels of the selected candidate genes at manipulated hind-paws. After CFA injection, at 1400 W, a highly selective inducible nitric oxide synthase (*iNOS*) inhibitor was administered regularly to elicit differences in CFA-induced pain behaviors and gene expression in HFD-fed mice. Results showed that HFD-fed mice were heavier (*p* < 0.001) and relatively hyperglycemic (*p* = 0.013) at baseline. HFD aggravated CFA-induced mechanical and thermal pain (mechanical: *p* = 0.0004, thermal: *p* = 0.003), showing prolonged hyperalgesic durations and reduced pain thresholds at multiple timepoints. HFD-influenced paws showed accentuated overexpression of pro-inflammatory cytokines and *iNOS* (RT-qPCR for *IL-1β*: *p* = 0.015, *IL-6*: *p* = 0.019, *TNF*: *p* = 0.04; ELISA for *iNOS*: *p* = 0.011). At 1400 W, exertion of analgesic effects (mechanical: *p* < 0.0001, thermal: *p* < 0.0001) but pro-inflammatory (RT-qPCR for *IL-1β*: *p* = 0.004, *IL-6*: *p* = 0.03, *TNF*: *p* = 0.04) were exerted on the inflamed paw on day 5 post-injection. In conclusion, short-term HFD aggravated CFA-induced inflammatory pain. Pharmacological inhibition of *iNOS* attenuated the CFA-induced pain in HFD-fed mice. Future research might uncover signaling pathways mediating such effects, potentially benefiting obese patients with chronic IP.

## 1. Introduction

Inflammatory pain (IP) develops at inflamed peripheral tissues when local inflammatory factors trigger intracellular signaling pathways in the peripheral terminals of the primary afferent neurons, hence altering the expression and function of receptor proteins and voltage-gated ion channels on the affected primary afferent neurons, rendering them hyperresponsive to mechanical and thermal stimulations [1]. Under conditions including osteoarthritis, rheumatoid arthritis and endometriosis, markers of inflammation were not only upregulated in patients with these disorders but also correlated with symptoms of pain [2]. Pain at the inflamed tissue could parallel increased levels of inflammatory mediators in the synovial fluid, including bradykinin, prostaglandins [3], tumor necrosis factor (*TNF*), interleukins [4], calcitonin gene-related peptide (CGRP) [5], and nerve growth factor-β (*NGF-β*) [6]. Other inflammatory factors, including prostaglandin E2 (PGE2) andnitric oxide (NO) have also been found to have a modulating effect on peripheral sensitization [7]. For example, PGE2 activates EP2 receptors on the peripheral terminals of high-threshold primary afferent neurons, hence reducing the firing threshold and improving signal transduction [7]. IP could be a troublesome symptom and a major threat to physical, mental, and social health. In terms of physical health, IP associated with rheumatoid arthritis predicted suboptimal treatment response and failure to achieve remission [8]. In terms of mental health, chronic pain and its severity predicted development of depression [9,10]. In terms of social wellbeing, inflammation-related pain could lead to increased social isolation and reduced social participation [11]. However, despite the heavy global disease burden associated with IP, the condition has remained a major therapeutic challenge due to the lack of safe and potent analgesic options. For example, paracetamol might sometimes be lacking in effectiveness [12,13], while chronic use of non-steroidal anti-inflammatory drugs (NSAIDs) could risk gastrointestinal and cardiovascular adverse events [14], and opioid analgesics could have addictive potential and respiratory suppressive effects [15]. Identification of novel therapeutic targets and discovery of safe and potent analgesic options remain important goals of translational biomedical research.

Inducible nitric oxide synthase (iNOS) has been known as one of the mediators of IP. Nitric oxide synthases (*NOSs*), including neuronal *nNOS* discovered on neurons, inducible *iNOS* found on activated macrophages, endothelial *eNOS* isolated on endothelial cells, and mitochondrial *mtNOS* found in mitochondria [16], are responsible for the biosynthesis of NO from L-arginine [17]. NO activates guanylyl cyclase (GC), leading to the conversion of guanosine triphosphate (GTP) to cyclic guanosine monophosphate (cGMP) [18]. Among the members of the *NOS* family, *iNOS* was characterized by its capability to synthesize large amounts of NO persistently for a long period of time [19]. Upregulation of *iNOS* at inflamed tissue has been observed following peripheral induction of inflammation [20,21]. Under a murine model of IP induced by complete Freund’s adjuvant (CFA), a pro-inflammatory solution containing inactivated *Mycobacterium tuberculosis* bacteria in mineral oil [22], *iNOS* inhibition partially reverted CFA-induced paw edema and mechanical hyperalgesia [23]. *iNOS* activity has been associated with both pro- and anti-nociceptive effects [24,25]. The NO/cGMP pathway primes hyperalgesia intradermally but reduces nociception in subcutaneous tissue [26]. With tissue inflammation, NO increased COX activity [27], resulting in increased biosynthesis of the pro-inflammatory PGE2, hence accounting for the pro-nociceptive effect [28]. On the other hand, multiple pathways underlying the peripheral analgesic effect of NO have been proposed [29].

According to a recent American guideline, 25–35% of the total calories in a healthy diet should come from fats [30]. If a diet consistently provides more than 35% of total calories by fats, it is referred to as a high-fat diet (HFD) [31]. Despite an ongoing trend among adults in the United States to adopt healthier dietary lifestyles, fats still accounted for 33.2% of their total energy intake in 2016 [32]. Under the influence of Westernization and low price of oils/fats, prevalence of dietary patterns rich in saturated fats was high in Southeast Asia and Europe [33]. In China, the prevalence of high-fat diet among the adult population had risen from less than 25% in 1991 to more than 67% in 2015, and the average percentage of total energy intake accountable to fat reached 35.2% [34]. HFD is often rich in linoleic acid and omega-6 polyunsaturated fatty acids (PUFAs) but relatively deficient in omega-3 PUFAs, including eicosapentaenoic acid (EPA) and docosahexaenoic acid (DHA) [35,36]. Adverse health outcomes associated with HFD have been well established, including type 2 diabetes mellitus (T2DM) [37], various malignancies [38], as well as problems with fetal development during pregnancy [39]. Even a short-term HFD could lead to reversible metabolic disturbances via adipokine imbalance or adipose tissue dysfunction [40]. Thus, both short-term and long-term HFD need to be studied. However, there has been no unified definition of short-term HFD regarding its duration. In general, the term has been utilized to describe experimental regimens lasting between 3 days and 4 weeks in rodent studies [41,42,43,44,45,46,47,48]. Durations not longer than 2 weeks could be chosen in order to reduce changes in metabolic physiology and body fat deposition patterns associated with obesity [45,49]. In spite of this, adverse physiological effects have been documented with less than 2 weeks of exposure. A 3-day HFD was able to increase body weight and fasting blood glucose [42]. In the central nervous system, ad libitum feeding with 60% HFD for 3–4 days resulted in disruption of hippocampal synaptic plasticity via the pro-inflammatory cytokine *IL-1β* [41]. Evidence of glial proliferation and neuronal injury could also be observed [50]. In a human study, 7-day exposure to 74% HFD was associated with heightened simple reaction times and compromised power of attention [51].

IP has been associated with increased dietary energy density, accounted for by dietary intake of cholesterol and fats, including saturated fatty acids, monounsaturated fatty acids and polyunsaturated fatty acids [52]. Unhealthy diet, along with other associated lifestyle factors, including physical inactivity and sedentary behavior, has been linked with the severity and persistence of chronic pain [53]. Long-term HFD has been shown to prolong and aggravate PPSP [54]. Liu et al. have also demonstrated that 40% caloric restriction for 6 weeks ameliorated HPI-induced mechanical and thermal hyperalgesia in non-obese rats, which correlated with decreased peri-incisional levels of pro-inflammatory cytokines *IL-1β*, *IL-6*, *TNF* and macrophage inflammatory protein-1 beta (*MIP-1β*), and increased peri-incisional level of the anti-inflammatory *IL-10* [55]. However, data from previous studies have indicated that HFD of shorter durations could still be sufficient to alter pain responses secondary to tissue inflammation [56,57]. The exact mechanisms underlying the effect of short-term HFD on tissue inflammation-induced pain remain unknown, as the short-course exposure to HFD might not result in overt diabetic phenotypes [58]. A 14-day HFD alone has been shown to increase *IL-1β* expression [59]. However, short-term HFD was not associated with significant changes in pre-injury pain thresholds, hence supporting an IL-1β-independent pathway mediating the effect of short-term HFD on inflammatory pain.

The current study aimed at (1) testing for any differences in behaviors of pain in an injection-based murine model of IP between animals with or without short-term high-fat dietary modification, and (2) identifying any potential role of *iNOS* in the development of HFD-associated differences in CFA-induced inflammatory pain behaviors. Differences in both mechanical and thermal hyperalgesia would be investigated in terms of pain behaviors. Since the differences in pain behaviors could most likely be attributed to an overall state of heightened tissue inflammation or known mediators of IP, as mentioned above, potential mediators would be screened from known mediators of IP. The mediating role would be tested by administering a selective *iNOS* inhibitor.

## 2. Results

### 2.1. CFA Injection Induced Inflammatory Pain Persisting for Days

**Figure 1:**
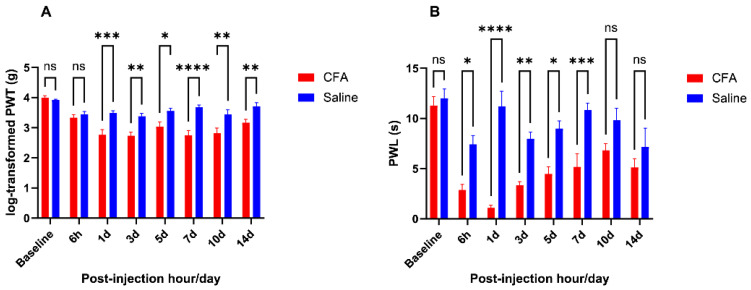
Comparing behavioral data from CFA and saline groups. (**A**): Log-transformed PWT from von Frey test (CFA group: *n* = 7; saline group: *n* = 8 for all time points); (**B**): PWL from Hargreaves’ test (CFA group: *n* = 6; saline group: *n* = 6 for all time points); ns (not significant) *p* ≥ 0.05, * *p* < 0.05, ** *p* < 0.01, *** *p* < 0.001, **** *p* < 0.0001, from two-way ANOVA with Šidák’s post hoc test.

Compared with saline controls, CFA injection resulted in mechanical and thermal hyperalgesia. Concerning the mechanical sensitivity measured as log-transformed PWT, two-way ANOVA showed a significant main effect for CFA versus saline group [F (1, 13) = 40.05, *p* < 0.0001] and a significant main effect for time point [F (7, 91) = 14.28, *p* < 0.0001] despite a significant interaction between animal group and time point variables [F (7, 91) = 4.64, *p* = 0.0002]. Regarding the thermal sensitivity measured as PWL, two-way ANOVA showed a significant main effect for CFA versus saline group [F (1, 10) = 77.41, *p* < 0.0001] and a significant main effect for time point [F (7, 70) = 9.39, *p* < 0.0001] despite a significant interaction between animal group and time point variables [F (7, 70) = 4.214, *p* = 0.0006]. The log-transformed PWT and PWL for mice in CFA and saline groups, at each tested time point, are summarized in Figure 1.

Compared with saline control, mice in the CFA group developed mechanical hyperalgesia lasting from post-injection day 1 to day 14, with the average magnitude of between-group difference peaking at day 5 (day 1: *p* = 0.0002, day 3: *p* = 0.0011, day 5: *p* = 0.016, day 7: *p* < 0.0001, day 10: *p* = 0.002, day 14: *p* = 0.009). Thermal hypersensitivity was demonstrated in the CFA group relative to the saline group from the 6th h after injection till the 7th post-injection day, with the maximum magnitude of between-group difference similarly obtained at day 5 (hour 6: *p* = 0.011, day 1: *p* < 0.0001, day 3: *p* = 0.009, day 5: *p* = 0.011, day 7: *p* = 0.0008).

**Figure 2:**
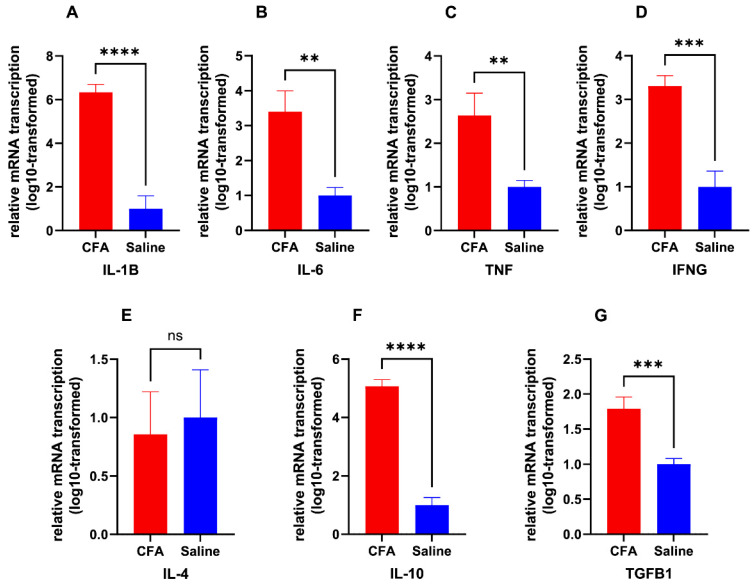
Relative transcription of inflammatory markers in CFA group. Log10-transformed 2^−ΔΔCt^ of transcribed mRNA on post-injection day 5, relative to saline group. (**A**): *IL-1β* (CFA group: *n* = 5, saline group: *n* = 6); (**B**): *IL-6* (CFA group: *n* = 7, saline group: *n* = 8); (**C**): *TNF* (CFA group: *n* = 6, saline group: *n* = 8); (**D**): *IFN-γ* (CFA group: *n* = 6, saline group: *n* = 6); (**E**): *IL-4* (CFA group: *n* = 5, saline group: *n* = 6); (**F**): *IL-10* (CFA group: *n* = 5, saline group: *n* = 8); (**G**): *TGF-β1* (CFA group: *n* = 6, saline group: *n* = 8). ns (not significant) *p* ≥ 0.05, ** *p* < 0.01, *** *p* < 0.001, **** *p* < 0.0001, from unpaired Student’s *t*-test.

The relative mRNA expression levels regarding common inflammatory marker genes, on the 5th day after CFA or saline injection are illustrated in Figure 2. The assayed genes included pro-inflammatory markers *IL-1β*, *IL-6*, *TNF*, and *IFN-γ*, as well as anti-inflammatory markers *IL-4*, *IL-10*, and *TGF-β1*. CFA group mice showed significantly elevated transcription of all assayed pro-inflammatory marker genes (*IL-1β*: *p* < 0.0001, *IL-6*: *p* = 0.0018, *TNF*: *p* = 0.004, *IFN-γ*: *p* = 0.0003). CFA also upregulated mRNA transcription for some anti-inflammatory cytokine genes, including *IL-10* and *TGF-β1* (*IL-10*: *p* < 0.0001, *TGF-β1*: *p* = 0.0006).

### 2.2. Short-Term HFD Induced Obesity and Aggravated CFA-Induced IP

**Figure 3:**
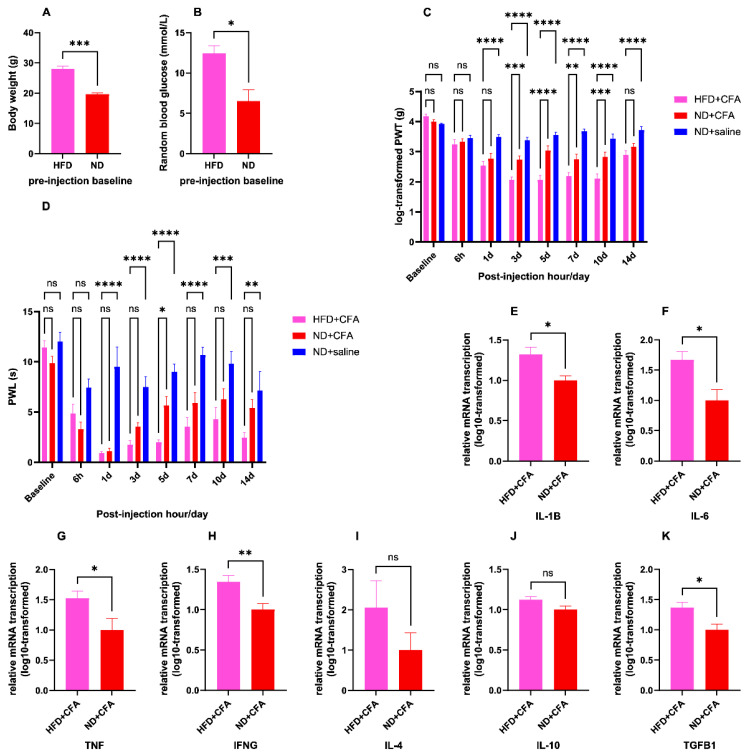
Effects of short-term high-fat diet. HFD + CFA: mice fed with HFD and injected with CFA; ND + CFA: mice fed with ND and injected with CFA; ND + saline: mice fed with ND and injected with normal saline. (**A**): body weight (in grams) of mice after being fed ad libitum with HFD or ND for 14 days (*n* = 4 per group); *** *p* < 0.001 from unpaired Student’s *t*-test. (**B**): random blood glucose (in mmol/L) of mice after being fed ad libitum with HFD or ND for 14 days (*n* = 4 per group); * *p* < 0.05 from unpaired Student’s *t*-test. (**C**): log-transformed PWT from von Frey test at various time points before and after CFA or saline injection, in mice with or without 14-day pre-injection dietary modification using ad libitum HFD (for all time points: *n* = 7 for HFD + CFA group, *n* = 7 for ND + CFA group, *n* = 8 for ND + saline group); ns (not significant) *p* ≥ 0.05, ** *p* < 0.01, *** *p* < 0.001, **** *p* < 0.0001, from two-way ANOVA with Šidák’s post hoc test. (**D**): PWL from Hargreaves’ test at various time points before and after CFA or saline injection, in mice with or without 14-day pre-injection dietary modification using ad libitum HFD (for all time points: *n* = 6 per group); ns (not significant) *p* ≥ 0.05, * *p* < 0.05, ** *p* < 0.01, *** *p* < 0.001, **** *p* < 0.0001, from two-way ANOVA with Šidák’s post hoc test. (**E**–**J**): Log10-transformed 2^−ΔΔCt^ of transcribed mRNA on day 5 after CFA injection, in mice with 14-day pre-injection dietary modification using ad libitum HFD, relative to normal diet group, in regards to common inflammatory marker genes of *IL-1β* ((**E**), HFD + CFA group: *n* = 5, ND + CFA group: *n* = 5), *IL-6* ((**F**), HFD + CFA group: *n* = 5, ND + CFA group: *n* = 7), *TNF* ((**G**), HFD + CFA group: *n* = 6, ND + CFA group: *n* = 6), *IFN-**γ*** ((**H**), HFD + CFA group: *n* = 6, ND + CFA group: *n* = 6), *IL-4* ((**I**), HFD + CFA group: *n* = 5, ND + CFA group: *n* = 5), *IL-10* ((**J**), HFD + CFA group: *n* = 6, ND + CFA group: *n* = 5), and *TGF-β1* ((**K**), HFD + CFA group: *n* = 6, ND + CFA group: *n* = 6). ns (not significant) *p* ≥ 0.05, * *p* < 0.05, ** *p* < 0.01 from unpaired Student’s *t*-test.

Figure 3 illustrates the effect of dietary modification with 14-day short-term high-fat diet before receiving CFA or saline injection on the affected mice, from metabolic, behavioral, and pathological aspects. Mice fed with HFD for 14 days since arrival had significantly higher body weights than their counterparts fed normal chow diet (ND group: mean weight = 19.68 g, HFD group: mean weight = 28.05, *p* = 0.0002), indicating the establishment of diet-induced obesity [60]. Besides, the random blood glucose levels of the HFD-fed mice averaged 12.43 mmol/L after 14 days of dietary modification, which was significantly higher than those of ND-fed mice, averaging 6.525 mmol/L at the same time point (*p* = 0.013).

Regarding the behavioral effects of short-term HFD on the susceptibility to CFA-induced IP, substitution of normal chow diet with HFD for 14 days did not result in any significant changes in the pain thresholds for either mechanical or thermal stimuli prior to the induction of IP (at baseline, log-PWT: *p* = 0.51, PWL: *p* = 0.42). However, after intra-plantar injection of CFA, HFD-fed mice demonstrated heightened mechanical hyperalgesia relative to the ND-fed group at post-injection days 3–10 (day 3: *p* = 0.0004, day 5: *p* < 0.0001, day 7: *p* = 0.002, day 10: *p* = 0.0001). Overall, the log-transformed PWT in the HFD-fed group was persistently lower than that of the control group receiving only saline injection and without dietary modification from post-injection day 1–14 (days 1, 3, 5, 7, 10, and 14: *p* < 0.0001 for each time point). Similarly, HFD-fed mice showed significantly aggravated thermal hyperalgesia on the 5th post-injection day, when compared to the ND-fed group (*p* = 0.014). Overall, the PWL in the HFD-fed group was persistently lower than the control group receiving only saline injection and without dietary modification from post-injection day 1–14 (day 1: *p* < 0.0001, day 3: *p* < 0.0001, day 5: *p* < 0.0001, day 7: *p* < 0.0001, day 10: *p* = 0.0001, day 14: *p* = 0.0012). Overall, considering the log-transformed PWTs, two-way ANOVA showed a significant main effect of animal group [F (2, 19) = 66.27, *p* < 0.0001] and a significant main effect of time point [F (7, 133) = 44.51, *p* < 0.0001] despite significant interaction between animal group and time point variables [F (14, 133) = 8.88, *p* < 0.0001]. Similarly, considering the PWLs, two-way ANOVA also showed a significant main effect of animal group [F (2, 15) = 69.41, *p* < 0.0001] and a significant main effect of time point [F (7, 105) = 17.51, *p* < 0.0001] despite a significant interaction between animal group and time point variables [F (14, 105) = 2.77, *p* = 0.0015].

Another pair of two-way ANOVA tests were performed to analyze the effect of diet group and time point on mechanical and thermal sensitivities, excluding the data from mice which received saline injection only. Concerning the mechanical sensitivity measured as log-transformed PWT, there was, still, a significant main effect for diet group [F (1, 12) = 24.22, *p* = 0.0004] and a significant main effect for time point [F (7, 84) = 42.79, *p* < 0.0001], despite a significant interaction between diet group and time point [F (7, 84) = 4.48, *p* = 0.0003]. Similarly, regarding the thermal sensitivity measured as PWL, there was a significant main effect for diet group [F (1, 10) = 14.14, *p* = 0.003] and a significant main effect for time point [F (7, 70) = 25.88, *p* < 0.0001], despite a significant interaction between diet group and time point [F (7, 70) = 3.056, *p* = 0.007].

In general, HFD-fed mice showed significantly elevated transcription of all assayed pro-inflammatory genes at the CFA-injected paws on day 5 after injection (*IL-1β*: *p* = 0.015, *IL-6*: *p* = 0.019, *TNF*: *p* = 0.04, *IFN-γ*: *p* = 0.0096). In contrast, *TGF-β1* was the only anti-inflammatory gene overexpressed in the HFD group (*p* = 0.014).

### 2.3. iNOS as an Upregulated IP Mediator Under HFD Influence

**Figure 4:**
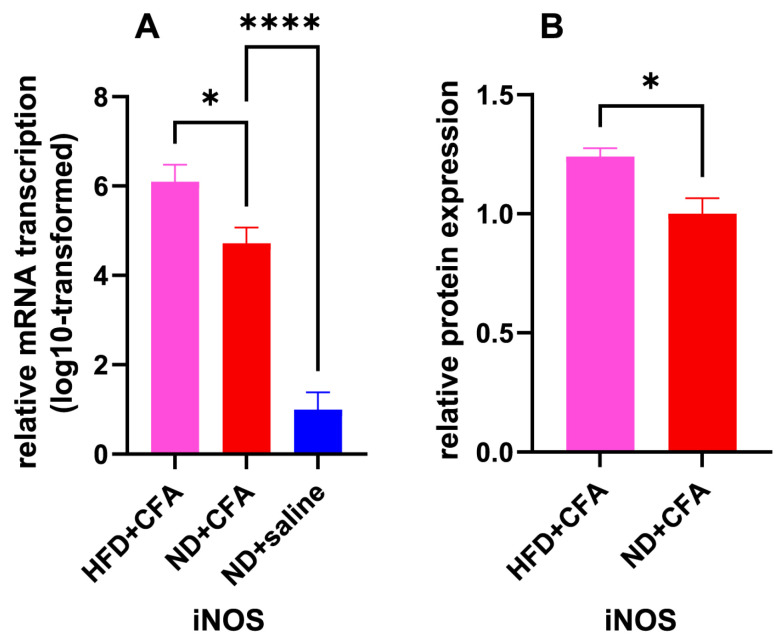
mRNA transcription and protein expression of iNOS gene under HFD and CFA influence. HFD + CFA: mice fed with HFD and injected with CFA; ND + CFA: mice fed with ND and injected with CFA; ND + saline: mice fed with ND and injected with normal saline. (**A**): Obtained from RT-qPCR assay, log10-transformed 2^−ΔΔCt^ of transcribed *iNOS* mRNA on post-injection day 5, in mice fed with HFD or ND and injected with CFA or normal saline, relative to normal diet—saline group (*n* = 5 for each group); * *p* < 0.05, **** *p* < 0.0001, from one-way ANOVA with Šidák’s post hoc test. (**B**): Obtained from ELISA, on post-injection day 5, protein expression level of *iNOS* in mice fed with HFD or ND and injected with CFA, relative to the ND + CFA group (*n* = 5 for each group). * *p* < 0.05, from unpaired Student’s *t*-test.

As one of the known mediators of IP, *iNOS* transcription in the CFA-injected paws on post-injection day 5 was significantly influenced by prior HFD feeding (one-way ANOVA: F (2, 12) = 49.95, *p* < 0.0001), with CFA-injected paws showing *iNOS* overexpression compared with saline controls and HFD-fed group showing further heightening of the CFA-induced overexpression (effect of CFA: *p* < 0.0001, effect of HFD: *p* = 0.04). Further ELISA confirmed significant increase in *iNOS* protein expression in the CFA-injected paws due to HFD feeding instead of standard chow diet (*p* = 0.011). The above findings are illustrated in Figure 4.

### 2.4. Analgesic but Pro-Inflammatory Effects of 1400 W

**Figure 5:**
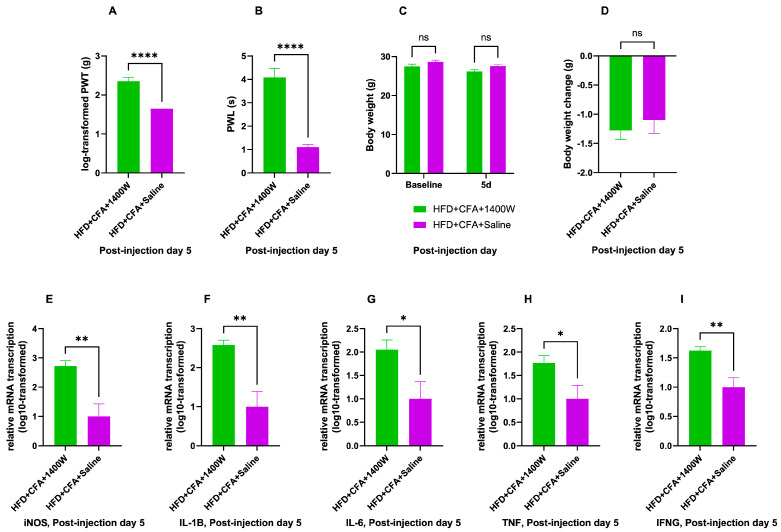
Effects of 1400 W on HFD-fed, CFA-injected mice. HFD + CFA + 1400 W: mice fed with HFD and injected with CFA, followed by post-injection intervention with 1400 W; HFD + CFA + Saline: mice fed with HFD and injected with CFA, followed by post-injection normal saline control. (**A**): log-transformed PWT from von Frey test on day 5 after CFA injection, in mice fed with HFD, with post-injection 1400 W intervention or saline control (*n* = 6 for each group); **** *p* < 0.0001, from unpaired Student’s *t*-test. (**B**): PWL from Hargreaves’ test on day 5 after CFA injection, in mice fed with HFD, with post-injection 1400 W intervention or saline control (*n* = 6 for each group); **** *p* < 0.0001, from unpaired Student’s *t*-test. (**C**): body weight (in grams) of mice after being fed ad libitum with HFD for 14 days and injected with CFA, followed by 1400 W intervention or saline control (n = 12 for each group); ns (not significant) *p* ≥ 0.05, from two-way ANOVA with Šidák’s post hoc test. (**D**): change in body weight (in grams) of mice since receiving CFA injection, in HFD-fed mice given with 1400 W intervention or saline control following CFA injection (*n* = 12 for each group); ns (not significant) *p* ≥ 0.05, from unpaired Student’s *t*-test. (**E**–**I**): Log10-transformed 2^−ΔΔCt^ of transcribed mRNA on day 5 after CFA injection, in mice fed with HFD, with post-injection 1400 W intervention or saline control, with regards to genes including *iNOS* (**E**), *IL-1β* (**F**), *IL-6* (**G**), *TNF* (**H**), and *IFN-γ* (**I**) (for all genes, *n* = 5 for each group); * *p* < 0.05, ** *p* < 0.01, from unpaired Student’s *t*-test.

In HFD-fed mice, intraperitoneal 1400 W or normal saline control was administered at regular intervals after receiving CFA injection until reaching experimental endpoints, with the resultant behavioral, body weight, and pathological changes presented in Figure 5.

Compared to intraperitoneal injection of saline controls, 1400 W administration resulted in significantly increased PWT and significantly increased PWL on the 5th day after CFA injection, compared to saline administration (PWT: *p* < 0.0001, PWL: *p* < 0.0001).

Comparison of body weight change between 1400 W and control groups at post-CFA-injectional day 5 has yielded unremarkable results. In general, the two-way ANOVA testing for the effect of time point and 1400 W on body weight yielded no significant main effect of 1400 W (F (1, 22) = 3.753, *p* = 0.53) on top of an interaction effect, which was also not significant (F (1, 22) = 0.40, *p* = 0.53). The main effect of time point was significant, (F (1, 22) = 74.74, *p* < 0.0001). This was consistent with a general trend of weight loss after CFA injection regardless of 1400 W treatment. Both groups demonstrated body weight loss without significant between-group differences (*p* = 0.53), given that there was no significant difference in body weight between both groups before receiving CFA injection (*p* = 0.16). There was no significant difference in body weight between both groups at post-injection day 5 either (*p* = 0.09).

Transcription levels of mRNA have been assayed after 5 days of 1400 W or saline control administration concerning the genes of *iNOS*, pro-inflammatory markers of *IL-1β*, *IL-6*, and *TNF*, as well as *IFN-γ*. Levels of mRNA transcription have been significantly upregulated for *iNOS* in the 1400 W relative to the control group (*p* = 0.006). Transcription of mRNA has also been heightened regarding all of the tested pro-inflammatory markers following 1400 W administration, compared to the control group (*IL-1β*: *p* = 0.004, *IL-6*: *p* = 0.03, *TNF*: *p* = 0.04, *IFN-γ*: *p* = 0.007).

## 3. Discussion

### 3.1. Short-Term HFD and IP

In the hind paws of chow diet-fed young adult mice, the current study has successfully adopted intra-plantar injection of CFA to induce mechanical and thermal hyperalgesia of magnitude and duration comparable to other published studies. Once-only local injection of CFA at a similar dosage typically produced an IP model in rodents lasting beyond 14–21 days [61,62]. In terms of the resultant change in expression of inflammatory marker genes after CFA injection, upregulation of pro-inflammatory cytokines *IL-1β*, *IL-6*, and *TNF* was consistent with the pro-inflammatory effects of CFA. *IL-10* and *TGF-β1* mRNA overexpression in the CFA group implied M2 macrophage polarization on post-injection day 5 and could potentially be induced by heightened *IL-6* and *IL-10* stimulation [63]. M2 polarization with concurrent upregulation of pro-inflammatory cytokines *IL-1β*, *IL-6*, and *TNF* was consistent with a chronic inflammatory state, as predictably induced by CFA [64].

With the introduction of short-term dietary modification using ad libitum lard-based HFD, the affected mice exhibited exaggerated thermal and mechanical hyperalgesia at the CFA-injected paws despite showing comparable pain thresholds before the injections. Hence, the current study created a context different from other previously published studies which elicited heightened mechanical and thermal sensitivity or spontaneous pain behaviors in animals under long-term HFD diets beyond 12 weeks and diet-induced painful diabetic neuropathy models [65,66]. Short-term HFD has been proven as effective in changing the behavioral profile of pain by prolonging the course of hyperalgesia induced by HPI without suppressing the pain thresholds pre-operatively [57]. The current study demonstrated exaggerated mechanical and thermal hyperalgesia at specific time points in addition to prolongation of the total duration of thermal hyperalgesia compared to control group with neither CFA injection nor dietary modification. Thermal hyperalgesia lasted from day 1 to day 14 in HFD-fed mice but disappeared after day 7 in ND-fed mice. Meanwhile, mechanical hyperalgesia was present from post-injection day 1 to day 14 among both ND-fed and HFD-fed groups, with day 14 being the experimental endpoint of the present study. The lack of significant prolongation of the course of mechanical hyperalgesia in the present study could be attributed to the long duration of mechanical hyperalgesia induced by CFA alone, which could extend well beyond the current experimental endpoint in higher doses of CFA [67]. Nonetheless, the HFD-fed mice demonstrated a significantly different overall pattern of mechanical and thermal sensitivity levels when compared to ND-fed mice after CFA injection, which was not limited to the fact that few time points with significant between-group differences were reflected by post hoc multiple comparison tests. In addition, The presence of statistically significant results illustrating the exaggeration of mechanical and thermal hyperalgesia could potentially be accounted for by the proper application of log-transformation on the log-normally distributed PWT data for proper usage of 2-way ANOVA, and the high statistical power of the Šidák’s post hoc test [68,69].

In terms of the inflammatory mechanisms underlying the HFD-induced effect, upregulation of mRNA transcription for *IL-1β*, *IL-6*, and *TNF* genes have been demonstrated by the present study. Previous experiments have demonstrated that short-term exposure to a high-glucose environment could increase lipopolysaccharide (LPS)-induced M1 macrophage polarization and activation in addition to upregulation of *IL-1β* and *IL-6* [70]. Elevated mRNA transcription of the *IL-1β*, *IL-6*, and *TNF* genes correlated with M1-like macrophage polarization in the tissue sample [71,72]. M1/M2 macrophage polarization ratio was linked to the pathogenesis mechanisms underlying countless conditions [73,74]. While *IFN-γ* could activate M1 macrophages, both *TNF* inhibitor etanercept and the anti-inflammatory cytokine *IL-4* could decrease the ratio of pro-inflammatory M1 to anti-inflammatory M2 macrophages [74]. Chen et al. summarized the mechanisms underlying macrophage polarization [63]. LPS could mediate M1 polarization by stimulating toll-like receptor (*TLR*) 4 and upregulating nuclear factor kappa B (*NF-κB*) via phosphatidylinositol 3-kinase/protein kinase B (*PI3K/AKT*) signaling cascade, hence releasing *IL-1β, IL-6, TNF*, and *iNOS* [75]. *NF-κB* could also upregulate the *PI3K/AKT* cascade and inhibit the mitogen-activated protein kinase (MAPK) in the MAPK/ERK pathway to therefore elevate the expression of *TNF* [76]. *IFN-γ* could induce M1 polarization via signal transducers and activators of transcription (*STAT*) 1, hence releasing *iNOS* as well. Cytokine receptors for *IL-1β*, *IL-6*, and *TNF* actives protein activator 1 (AP-1), leading to augmented release of these cytokines as well as *iNOS*. On the other hand, *IL-10, IL-4*, and fatty acid stimulated M2 polarization and resulted in release of *IL-10* as well as other anti-inflammatory factors [63,71].

### 3.2. iNOS as the Mediator of HFD-Induced Aggravation of IP

As a known mediators of IP, *iNOS* showed overexpression associated with CFA and the additional HFD feeding in the present study. Upregulation of *iNOS* expression after CFA injection has also been observed in previous studies. For example, CFA injection in the intervertebral disc resulted in localized *iNOS* overexpression at 2 weeks after injection, which was the earliest post-injection time point tested in the study [77]. Under a pro-inflammatory condition, *iNOS* overexpression was often coupled with upregulation of other pro-inflammatory cytokines, including *IL-1β* and *IL-6*, as well as M1 macrophage polarization induced by *NF-κB* [63]. In addition, a positive feedback loop has been proposed between *iNOS* and *NF-κB*, as the NO produced by *iNOS* to inhibit sirtuin 1 (*SIRT1*) via S-nitrosylation, hence reducing the inhibitory deacetylation of p53 and p65 subunits of *NF-κB* [78]. *iNOS* overexpression could be maintained by active walking movements by the injected mice during non-sedated hours, as repetitive mechanical stress could stimulate localized *iNOS* expression in multiple body sites [79,80]. Although the in vivo activity of the overexpressed iNOS could be suppressed post-translationally [81,82], the significant behavioral and molecular changes following 1400 W treatment relative to saline control in the current study supported the local presence of biologically active *iNOS* in HFD-fed mice after CFA injection.

Tracing upstream evidence has shown that the expression and activity of *iNOS* could be regulated by multiple signaling pathways. The *NF-κB* signaling cascade was among the most commonly discussed, as it has successfully induced *iNOS* expression on multiple cell types [76]. As a part of inflammatory response, *IL-1β* promoted degradation of *IκBα*, the key inhibitor in the *NF-κB* pathway, and resulted in overexpression of the p65 subunit of *NF-κB* in the nucleus [83]. As a result, *IL-1β* could upregulate protein expression of *iNOS* and *COX-2* [84]. Another mediator of *NF-κB* upregulation was *TLR2*, which could also mediate *TNF* and *IL-1β* overexpression [85]. Meanwhile, poly(ADP-ribose) polymerase (*PARP*) could also upregulate *iNOS* activity via an *NF-κB*-independent pathway [80]. In order to achieve inhibitory effects, AMP-activated protein kinase (*AMPK*) could inhibit the nuclear translocation of *STAT3*, and promote cytoplasmic *STAT3* Ser727-phosphorylation, hence downregulating *iNOS* expression [86]. Under conditions with tissue ischemia, hypoxia-inducible factor 1-alpha (*HIF-1α*), as one of the earliest markers of cellular hypoxia, stimulated *iNOS* activity to produce nitric oxide [87]. As nitric oxide reacts with oxygen to form peroxy-nitrite, the resultant oxidative environment would inhibit *HIF-1α* activity, hence completing a negative feedback loop [88]. *HIF-1α* was also implicated in inflammatory conditions, including rheumatoid arthritis, where upregulations of *HIF-1α* and *iNOS* were both seen in synovial fluids [89]. Finally, *iNOS* expression could be influenced by other inflammatory cytokines in various cell types and tissues. Previous studies have shown that expression of *iNOS* and other pro-inflammatory cytokines, including *IL-1β*, *IL-6* and *TNF*, could be induced by a cytokine cocktail containing *IFN-γ* [90]. Another cytokine cocktail comprising of *IL-1β, TNF*, and *IFN-γ* could also upregulate *iNOS* activity [91]. *IFN-γ* could augment the stimulatory effect of LPS and induce a 30-increase in NO release by the stimulated macrophages [92]. Even *TNF* alone could play a positive, pivotal role in determining local *iNOS* expression levels [72]. The effects of *TNF* could be mediated via multiple pathways, possibly independent of *NF-κB* signaling, for example, necroptosis via receptor interacting protein 1 (*RIP1*) [93]. In terms of mechanical stimulatory pathways, physical force application has demonstrated M1-polarizing effects on macrophages and resulted in overexpression of M1-associated cytokine *TNF* [72]. Such an effect was likely mediated by the *NF-κB* signaling cascade, as *NF-κB* pathway inhibitors could reverse the upregulated *iNOS* expression induced by mechanical stress [80].

The current study further demonstrated upregulated mRNA transcription and protein expression of the *iNOS* gene in HFD-fed mice on day 5 after CFA injection at the affected plantar paw skin tissue. This was consistent with previous studies, which showed that the expression of *iNOS* could be induced in tissues of cholesterol-fed mammals [94]. Tissue specimens could yield higher levels of *iNOS* mRNA and protein in diabetic human patients than non-diabetic patients, resulting in an oxidative environment to relieve tissue ischemia, as supported by attenuated HIF-1α expression levels in samples from diabetic patients, which was further correlated negatively with the serum hemoglobin A1c levels [88]. On the cellular level, LDL-cholesterol could upregulate *iNOS* activity in macrophages [94]. Recently, leptin, an adipokine, has become a common research field of interest due to its connection with inflammation and inflammatory disorders [93]. Generally upregulated in obese humans [67], leptin could promote M1 polarization of macrophages and correlated with overexpression of pro-inflammatory cytokines, which were downstream effects reversible by a *TNF/RIPK* pathway inhibitor [93]. *IL-32* presented as another molecule of interest with its pro-inflammatory effects via the *NF-κB* and *TNF* pathways and generalized overexpression in obese humans [95]. *IL-32* has also demonstrated efficacy in inducing *iNOS* mRNA transcription and protein expression [96]. Besides, hormonal influence might be responsible for HFD-induced augmentation of *iNOS* overexpression. Estradiol was found in higher serum levels among men with obesity [97]. Estradiol could, via cadherin-11, upregulate the expression of *iNOS* and *IL-6* induced by *TNF* [98].

The current study has demonstrated the analgesic effect of a systemically administered specific *iNOS* inhibitor in HFD-fed young adult mice for CFA-induced IP. With regards to IP, prophylactic inhibitors of *iNOS* have demonstrated efficacy in decreasing pain in zymosan injection-induced arthritis [99]. It has been hypothesized that *iNOS* is associated with IP because its NO production would provide oxidative stress and lead to mitochondrial dysfunction [86]. *iNOS* could also lead to *TRPV1* activation, hence mediating thermal hyperalgesia [100]. Estradiol-induced cadherin-11 activity could also mediate mechanical hyperalgesia in animal models of IP [98].

The analgesic effects of 1400 W were coupled with pro-inflammatory effects under the current findings. Subsequent to 1400 W injection, the mRNA expression of *iNOS*, along with other pro-inflammatory cytokine genes, have raised significantly compared to their counterparts receiving saline injections. Under the process of wound healing, both pro-inflammatory and anti-inflammatory effects of *iNOS* have been proposed. This could either be beneficial and promote healing or be destructive and promote fibrosis [101]. NO has successfully suppressed *iNOS* expression in neutrophils and microglia, hence suggesting a negative feedback loop revolving around *iNOS* [102]. It has been demonstrated that *IFN-γ* from activated T cells could activate myeloid suppressor cells to inhibit further T cell function and proliferation via extracellular NO signaling generated by *iNOS* [103]. *IFN-γ* also promoted the differentiation of monocytes into CD86− regulatory macrophages, which phagocytized both CD4+ T cells and CD8+ T cells via its expression of *iNOS*, hence resulting in an anti-inflammatory effect [104]. It has been proposed that intracellular NO formation could also inhibit caspase-1 activity and suppress *IL-1β* levels [105]. NO could also induce S-nitrosylation of the cellular inhibitor of apoptosis protein 1 (*cIAP1*), hence preventing ubiquitin-mediated degradation of *RIP1* and resulting in inhibitory feedback on *TNF/RIPK* signaling and the necroptosis pathway [106]. The negative feedback mechanism could also be mediated by *IL-32*, which was inducible by *iNOS* inhibitors [96]. Recently, RNA sequencing has demonstrated an inhibitory effect of *iNOS* on the activation of immune pathways, especially those related to chemokine and *IFN-γ* and *NF-κB* signaling [107]. Overall, *iNOS* deficiency could mediate upregulation of *IL-1β* and *IL-6*, possibly without disturbing the transcriptional profile of *TNF* [108]. mRNA transcription was only mildly upregulated following 1400 W treatment in the current study, to a small magnitude less than 10-fold, whereas the upregulation of *IL-1β* transcription reached a 37-fold magnitude. In fact, obesity, itself indicating a chronic state of low-level inflammation, has been associated with generally elevated levels of pro-inflammatory cytokines, including *IL-6*, *TNF*, as well as *iNOS* detectable in serum [109]. Since high-fat diet augmented the CFA-stimulation *TNF* mRNA transcription in the current study, there should exist an *iNOS*-independent pathway to the accentuated inflammation associated with HFD. The mRNA transcription levels of inflammatory cytokines were not tested before CFA injection. However, the upregulation of mRNA transcription, if any, has proven insufficient to lowering the pain thresholds in HFD-mice before CFA injection.

Summing up, it was unsurprising that intra-plantar *iNOS* expression could be upregulated by CFA injection, as well as the additional HFD feeding due to the underlying inflammatory state, just as iNOS was implicated in other inflammatory or autoimmune disorders. Among the potential upstream regulators of *iNOS*, including *NF-κB*, *PARP*, *AMPK*, *HIF-1α*, and *TNF/RIPK* pathways, the latter two, as well as estradiol hormone, emerged as the candidates most likely to mediate the upregulation of *iNOS* activity associated with HFD on CFA-influenced paw tissue, based on the existing available evidence reviewed. Through experimentation with 1400 W, the current study supports the argument for an anti-inflammatory but pro-nociceptive effect associated with *iNOS* in the established in vivo model. Decoupling of *iNOS* activity with expression of pro-inflammatory cytokines and *iNOS* itself spoke against the involvement of *NF-κB* signaling pathway in mediating the *iNOS*-associated effects. In contrast, the current study supported a suppressive effect of *iNOS* on *NF-κB* signaling. *IL-32* also served as a potential mediator for the observed effects associated with HFD and 1400 W. However, further research would be needed in order to better characterize its effects on the immune pathways under various contexts, since both pro-inflammatory and anti-inflammatory effects of *IL-32* have been previously demonstrated [95]. The analgesic effects of 1400 W could be mediated through multiple pathways, including attenuation of oxidation stress in mitochondria and reduction in *TRPV1* activity, both of which could be active at peripheral body sites. Although 1400 W did not significantly alter body weight change after CFA injection, it remains unclear whether localized or generalized metabolic effects of the systemically administered 1400 W could partially mediate its analgesic effects, as the pathways involved were complex and beyond the scope of the current study.

### 3.3. Clinical Implications of iNOS-Associated Findings

In the future, there might be a role for pharmacological adjuncts with inhibitory effects on *iNOS* in the treatment of IP conditions for patients with obesity or high-fat dietary habit under refractory scenarios or suboptimal pain control using routine analgesic regimens. Multiple substances have recently been shown to have direct or indirect suppressive effects on *iNOS* activity within in vivo or in vitro studies over the past decades [71,110,111,112,113,114,115]. Cannabidiol has also demonstrated efficacy in downregulating *NF-κB* nuclear translocation the expression of *iNOS*, *IL-1β*, and *COX-2*, as well as the production of NO [116,117]. Melatonin demonstrated a suppressive effect on LPS-stimulated *iNOS* expression by in vitro macrophages with a dose-response effect [75], and 5-hydroxytryptophan has demonstrated suppressive effects on LPS-induced expression of *iNOS* along with *IL-6* and *COX-2*, via the *MAPK/ERK* pathway [118]. Locally administered magnesium sulphate could also suppress *IL-1β* and *iNOS*, resulting in attenuation of HPI-induced thermal hyperalgesia [119]. Omega-3 fatty acids and curcumin, both commonly used dietary supplements, could downregulate *iNOS* and *COX-2* expression with synergistic effects [120].

### 3.4. Limitations

Only young, male, mice were used in the present study. Aged mice might have demonstrated higher mechanical and thermal thresholds for pain, due to higher spinal cortisol levels suppressing the *NF-κB* signaling cascade [121]. Meanwhile, sex did not account for any significant differences in pain thresholds [121].

Regarding the short-term HFD feeding plan, it remains uncertain whether isoenergetic replacement of dietary carbohydrate intake could aggravate CFA-induced IP. In addition, only a lard-based HFD has been used in the current study, where the proportions of various fatty acids remain fixed. In a diet with 20% weight accounted for by fats, higher proportion of saturated fatty acid was associated with lower muscle glucose content, while the proportions of cis fatty acid, trans fatty acid, or unsaturated fatty acid did not significantly alter glucose metabolism [122]. Omega-3 polyunsaturated fatty acid supplementation, in particular, had moderate therapeutic effects on chronic IP as it significantly decreased pain scores on average [123]. The ketogenic diet is another example of a high-fat, low-carbohydrate plan, which has shown efficacy in downregulating baseline sensitivity to thermal pain [124].

Finally, the analgesic effect of 1400 W was only tested at a single time point days after CFA injection. There might be *iNOS*-independent pathways mediating the HFD-associated aggravating effects at earlier time points. In another study, *iNOS* correlated with pain behaviors at chronically inflamed joints, but predicted attenuated pain during the acute phase [125]. Genetic knockout of *iNOS* in mice reduced intra-plantar zymosan-induced early thermal hyperalgesia [126]. Further experimentation would be needed to profile the time course of *iNOS* upregulation following CFA injection in HFD-fed mice, and to identify any pro-nociceptive and anti-inflammatory effects of *iNOS* present in the acute phase or at later time points.

## 4. Materials and Methods

### 4.1. Animal Husbandry and Feeding

**Figure 6:**
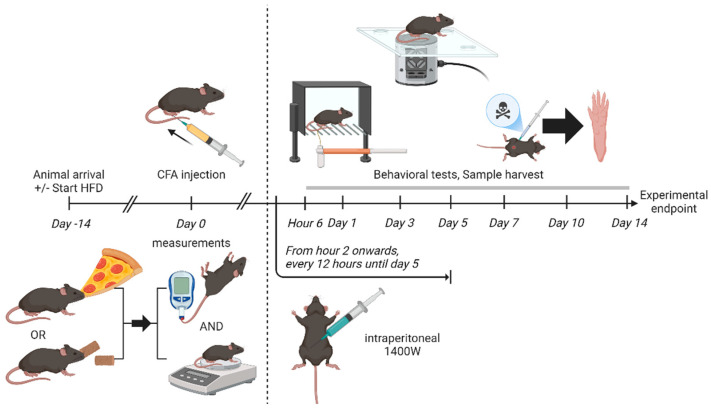
Experimental time points.

Male C57BL/6J mice (The Jackson Laboratory, Bar Harbor, ME, USA) at 8 weeks of age were kept in groups of 4 per individually-ventilated, autoclaved, cage, with ad libitum drinking water provision and 24-h light-dark cycle. A per-group sample size of 4 would be sufficient to illustrate a statistically significant mean difference of 33.3% with 80% power and *p* < 0.05. Depending on the experimental group as assigned by random, mice were fed with either lard-based HFD (D12492, Research Diets, Inc., New Brunswick, NJ, USA) or normal chow diet (irradiated version, LabDiet^®^ 5053, Land O’Lakes, Inc., Arden Hills, MN, USA) ad libitum from their arrival 2 weeks before CFA injection till euthanasia, without any change of diet during the whole period. The lard-based high-fat diet provided 60% of the total calories through fat, while only 13% of total calories were stored as fat in the normal chow diet. Subsequent experimental time points are shown in Figure 6.

### 4.2. Random Blood Glucose Testing

Random blood glucose testing was performed at a specified fixed time point. Blood samples were collected by tail-tip amputation on a test strip and the glucose levels were measured by a glucometer (OneTouch^®^ SelectTM Plus, LifeScan Europe, Zug, ZUG, Switzerland).

### 4.3. Euthanization and Tissue Isolation

Animals were sacrificed with overdose of intraperitoneal sodium pentobarbital (100–150 mg/kg, Dorminal 20%, Alfasan International B.V., Woerden, Holland) with reference to the American Veterinary Medical Association (AVMA) Guidelines for the Euthanasia of Animals. Animals which had suffered from injury due to fighting behaviors were prematurely culled. Plantar skin of the injured hind paw, including the adhered subcutaneous fat, were harvested immediately after euthanasia. The sample was frozen using liquid nitrogen and stored under −80 °C for further molecular assays.

### 4.4. CFA Injection Model of IP

Mice were anesthetized with isoflurane (5% for induction, 1.5–2% for maintenance, cat. 2024070701, RWD Life Science Co., LTD, Sugar Land, TX, USA) in 1 L/min O_2_ delivered via a nose cone by a veterinary inhalation anesthesia machine (RSA Small Vet Anesthesia Machine, RWD Life Science Co., LTD, Sugar Land, TX, USA). A total of 20 µL CFA (Mycobacterium tuberculosis; F5881, Sigma-Aldrich, Inc., St. Louis, MO, USA), without prior emulsification, was injected subcutaneously with a gas-tight syringe (25 µL gas phase tip microinjector, Shanghai Gaoge Industry and Trade Co., Ltd., Shanghai, China) into the plantar side of the left hind paw from the first interdigital space. The needle was held inside the subcutaneous space for 20 s to allow diffusion of the viscous CFA across the whole plantar subcutaneous space before withdrawal. The control group of mice received a sham injection procedure using steam-autoclaved normal saline instead of CFA, with all other steps changed.

### 4.5. Drug Administration

1400 W (sc-3564A, Santa Cruz Biotechnology, Inc., Dallas, TX, USA), a highly selective iNOS inhibitor, dissolved in normal saline, was injected intraperitoneally at a dosage of 10 mg/kg [127,128] every 12 h, from 2 h after CFA injection to experimental endpoint. Animals in control group received intraperitoneal normal saline injections with the same volume and at the same time points.

### 4.6. Behavioral Tests

All behavioral tests were performed with the experimenter blinded to treatment allocation. Mechanical and thermal nociceptive thresholds were assessed using the manual von Frey test [129] and the Hargreaves’ test [130], respectively, with adequate pre-measurement acclimatization. Time points for performing behavioral tests included pre-procedural baseline, at the 6th h after the procedure, as well as post-procedural days 1, 3, 5, 7, 10, and 14.

#### 4.6.1. Manual Von Frey Test 

Mice were placed in separate plexiglass boxes on a stainless-steel mesh floor for 90 min to acclimatize to the environment. The boxes were covered by a black piece of cloth on all sides except for the bottom to minimize any light-induced psychological distress. A series of Semmes–Weinstein monofilaments (cat. 514000-20C, DanMic Global, LLC, San Jose, CA, USA) with calibrated target forces (0.008, 0.02, 0.04, 0.07, 0.16, 0.40, 0.60, 1.0, 1.4, in grams), were used to test the sensitivity of the plantar surface of the hind paw towards tactile mechanical stimuli. Choices of filaments and derivation of paw withdrawal thresholds (PWT) were based on the up-and-down method. Triplicated PWT measurements per mice were averaged geometrically to yield the timepoint-specific mean PWT.

#### 4.6.2. Hargreaves’ Test 

Animals were placed in a plexiglass chamber on a transparent glass surface. The plantar test was carried out with a paw analgesia meter (IITC Life Science, Inc., Woodland Hills, CA, USA). A focused, adjustable, radiant heat light source (37300-002, Ugo Basile SRL, Italy) beneath a glass floor would be directed onto the plantar surface of a hind paw. The heat source was calibrated with an infrared heat-flux radiometer (37300-001, Ugo Basile SRL, Gemonio, VA, Italy). Three individual readings of heat-induced paw withdrawal latency (PWL) were taken for each tested hind-paw with an interval of at least 5 min and averaged arithmetically as the average mouse-specific, timepoint-specific PWL.

### 4.7. Molecular Assays

In the present study, messenger ribonucleic acid (mRNA) expression levels in non-fixed murine tissue samples were quantified by real-time polymerase chain reaction (RT-qPCR) assay, and protein expression levels by enzyme-linked immunosorbent assay (ELISA). The experimental group assignment corresponding to each assayed animal sample was blinded from the researcher performing the molecular assays.

#### 4.7.1. RT-qPCR Assay

Frozen, non-fixed, hind paw skin samples were homogenized by a handheld disperser (POLYTRON^®^ PT 1200 E, Kinematica AG, Malters, CH, Switzerland) for subsequent mRNA using a guanidinium thiocyanate and phenol-based protocol (cat. TR 118, Molecular Research Center, Inc., Cincinnati, OH, USA). The resultant mRNA was dissolved in 20 µL of steam-autoclaved, double-distilled water. The mRNA concentration was tested by light absorptiometry using a microplate reader (Epoch 2 Microplate Spectrophotometer, Santa Clara, CA, USA). Readings yielding 260/280 ratio less than 2 were discarded. Subsequently, the mRNA underwent reverse transcription reaction using RT MasterMix (cat. RR036A, Takara Biomedical Technology Co., Ltd., Beijing, China) in a thermal cycler (cat. 186-1096, Bio-Rad Laboratories, Inc., Hercules, CA, USA). RT-qPCR was performed with a real-time PCR system (cat. 05015243001, Roche Diagnostics International AG, Rotkreuz, Switzerland) under standard TB green protocol (cat. RR420, Takara Biomedical Technology Co., Ltd., Beijing, China). Primers used in this study are listed in Table 1 below, with *RPLP0* being used as the housekeeping gene (Integrated DNA Technologies, Inc., Coralville, IA, USA). RPLP0 was chosen due to its stability under obesity [131,132] and inflammatory conditions [133,134]. Results with Ct values greater than 40 were considered negative. The 2^−ΔΔCt^ method was used for relative quantification [135].

#### 4.7.2. ELISA

Frozen paw skin was homogenized in a lysis buffer solution (cat. 9803S, Cell Signaling Technology, Inc., Danvers, MA, USA) with protease inhibitor (cat. 04693159001, Roche Diagnostics AG, Rotkreuz, Switzerland) and phosphatase inhibitor (cat. 04906845001, Roche Diagnostics AG, Rotkreuz, Switzerland). The extracted protein concentration from each sample was determined using a Bradford-based protein assay, correlating the light absorption at 595 nm wavelength of sample solutions with a linear standard curve obtained from a set of standard bovine serum albumin (cat. BSA-50, Rockland Immunochemicals, Inc., Limerick, PA, USA) solutions at concentrations of 8, 4, 2, 1, 0.5, 0.25, 0.125, and 0.0625 g/L. Samples were diluted to a uniform total protein concentration with lysis buffer solution and stored at −80 °C for further ELISA experiments, which was performed using an *iNOS*-specific kit (cat. KBR-PD4511S, Shanghai Keborui Biotechnology Co., Ltd., Shanghai, China). The *iNOS* concentration in each sample was averaged from triplicated results from interpolation of measured optical densities against a linear standard curve.

### 4.8. Statistical Analyses

Body weight and blood glucose measurements were compared by unpaired Student’s *t*-test in scenarios with only a single time point, and by 2-way analysis of variance (ANOVA) with Šidák’s post hoc test for longitudinal follow-up of the same batch of mice over multiple time points.

For behavioral tests, the measurement of different experimental groups at each time point was compared by Šidák’s post hoc test based on 2-way ANOVA results. PWTs from von Frey test were log-transformed before performing ANOVA due to their assumed log-normality based on the well-accepted Weber’s law, as proposed by Mills et al. [69]. Hence, the plotted reduction of the log-transformed PWT per unit would represent a 10-fold decrease in the PWT, measured in grams or micronewtons.

For molecular assays, 2^−ΔΔCt^ for each gene from RT-qPCR assay and absorbance of each protein species from ELISA were log-transformed before using them to compare between experimental groups using unpaired Student’s *t*-test or one-way ANOVA with Šidák’s post hoc test, depending on the number of experimental groups present. *p* < 0.05 was considered statistically significant. All statistical analyses and graphs were made using GraphPad Prism (version 9.4.0 for Windows, GraphPad Software, Boston, MA USA).

## 5. Conclusions

Intra-plantar injection of CFA induced mechanical and thermal hyperalgesia persisting up to 14 days after injection. CFA-induced pain behaviors paralleled upregulation of pro-inflammatory cytokines, which could be mediated by *NF-κB* signaling. Short-term ad libitum HFD feeding for 14 days resulted in significant weight gains and relative hyperglycemia resembling obesity. HFD prolonged CFA-induced thermal hyperalgesia and further reduced nociceptive thresholds at selected few time points. Overexpression of pro-inflammatory cytokines could be accounted by M1 polarization of macrophages associated with HFD. This study identified *iNOS* as a vital molecule linking aggravated CFA-induced tissue inflammation and augmentation of CFA-induced mechanical and thermal hyperalgesia in HFD-fed mice. *iNOS* was locally upregulated by short-term HFD on the 5th day after CFA injection. Experimentation with 1400 W suggested that the HFD-induced *iNOS* expression mediated pro-nociceptive but anti-inflammatory effects.

Some of The above findings could potentially become clinically relevant. Firstly, the currently adopted in vivo model showed that high-fat dietary habit and diet-induced obesity could lead to worse IP when triggered by the appropriate etiology, which was intra-plantar CFA injection in the current study. Secondly, these patients might require pharmacological or non-pharmacological adjuncts in order to achieve satisfactory pain control. Options with inhibitory effects on *iNOS*, regardless of the novelty of its discovery, could potentially complement routinely used anti-inflammatory drugs to improve pain control, while balancing the pro-inflammatory effects of *iNOS* inhibition by the anti-inflammatory regimen already prescribed.

Future efforts could be directed to verifying the roles of these pathways *HIF-1α*, leptin/*TNF/RIPK* and estradiol/cadherin-11in mediating the HFD-induced aggravation of IP, in addition to studying how HFD increases the activity of these pathways, and whether *iNOS* mediates the aggravated pain in other time points in HFD-fed animals.

## Figures and Tables

**Table 1:** RT-qPCR primers.

Gene	Forward			Reverse		
	Sequence	Ln	Tm (°C)	Sequence	Ln	Tm (°C)
*IFN-γ*	AAATCCTGCAGAGCCAGATTAT	22	54.1	GCTGTTGCTGAAGAAGGTAGTA	22	54.5
*IL-10*	GAGGCGCTGTCATCGATTT	19	55.5	CACCTTGGTCTTGGAGCTTATT	22	54.9
*IL-1β*	CCACCTCAATGGACAGAATATCA	23	54.4	CCCAAGGCCACAGGTATTT	19	55.1
*IL-4*	TTGAGAGAGATCATCGGCATTT	22	54.0	CTCACTCTCTGTGGTGTTCTTC	22	54.9
*IL-6*	AAGACAAAGCCAGAGTCCTTC	21	54.7	CCTTCTGTGACTCCAGCTTATC	22	54.8
*iNOS*	GTCTGCATGGACCAGTATAAGG	22	54.9	TTCTTCAGAGTCTGCCCATTG	21	54.8
*RPLP0*	AGAAACTGCTGCCTCACATC	20	55.2	CAGCAGCTGGCACCTTATT	19	55.6
*TGF-β1*	GCAACAATTCCTGGCGTTAC	20	54.7	GTATTCCGTCTCCTTGGTTCAG	22	55.0
*TNF*	CCTCTTCTCATTCCTGCTTGT	21	54.5	TGGGAACTTCTCATCCCTTTG	21	54.6

Abbreviations: Ln, nucleotide length; Tm, melting temperature.

## Data Availability

The original contributions presented in this study are included in the article. Further inquiries can be directed to the corresponding author(s).

## Abbreviations

The following abbreviations are used in this manuscript:

CFA	Complete Freund’s adjuvant
HFD	High-fat diet
iNOS	Inducible nitric oxide synthase
